# Cellular Composition of Cerebrospinal Fluid in HIV-1 Infected and Uninfected Subjects

**DOI:** 10.1371/journal.pone.0066188

**Published:** 2013-06-18

**Authors:** Emily L. Ho, Rollie Ronquillo, Hermann Altmeppen, Serena S. Spudich, Richard W. Price, Elizabeth Sinclair

**Affiliations:** 1 Department of Neurology, Harborview Medical Center, University of Washington, Seattle, Washington, United States of America; 2 Department of Immunology and Microbiology, Rush University Medical Center, Chicago, Illinois, United States of America; 3 Institute of Neuropathology, University Medical Center Hamburg-Eppendorf, Hamburg, Germany; 4 Department of Neurology, Yale University, New Haven, Connecticut, United States of America; 5 Department of Neurology, University of California San Francisco, San Francisco, California, United States of America; 6 Division of Experimental Medicine, University of California San Francisco, San Francisco, California, United States of America; University of Cape Town, South Africa

## Abstract

In order to characterize the cellular composition of cerebrospinal fluid (CSF) in a healthy state and in the setting of chronic pleocytosis associated with HIV-1 (HIV) infection, multi-parameter flow cytometry was used to identify and quantitate cellular phenotypes in CSF derived from HIV-uninfected healthy controls and HIV-infected subjects across a spectrum of disease and treatment. CD4+ T cells were the most frequent CSF population and the CD4:CD8 ratio was significantly increased in the CSF compared to blood (p = 0.0232), suggesting preferential trafficking of CD4+ over CD8+ T cells to this compartment. In contrast, in HIV-infection, CD8+ T cells were the major cellular component of the CSF and were markedly increased compared to HIV-uninfected subjects (p<0.001). As with peripheral blood, the CSF CD4:CD8 ratio was reversed in HIV-infected subjects compared to HIV-uninfected subjects. Monocytes, B cells and NK cells were rare in the CSF in both groups, although absolute counts of CSF NK cells and B cells were significantly increased in HIV-infected subjects (p<0.05). Our studies show that T cells are the major cellular component of the CSF in HIV-infected and uninfected subjects. The CSF pleocytosis characteristic of HIV infection involves all lymphocyte subsets we measured, except for CD4+ T cells, but is comprised primarily of CD8+ T cells. The reduced proportion of CD4+ T cells in the CSF may reflect both HIV-related peripheral loss and changes in trafficking patterns in response to HIV infection in the central nervous system.

## Introduction

HIV-1 (HIV) infection is frequently accompanied by cerebrospinal fluid (CSF) pleocytosis that occurs early in infection and largely resolves with antiretroviral therapy (ART) [1], [2], [3], [4], [5], [6]. The cellular composition of this pleocytosis has not been fully defined, particularly in patients with treated HIV infection and in comparison to HIV-uninfected individuals. Flow cytometry provides a powerful method for elucidating multiple cell characteristics in fluids, such as CSF [7], [8], [9], [10], [11]. Advances in polychromatic flow cytometry have increased the number of markers that can simultaneously be applied to a sample [12], allowing a more comprehensive analysis of CSF, with its relatively low number of cells, than was possible in earlier studies [8], [10]. Flow cytometry has been used to evaluate CSF B and T cell subsets in various neurological conditions [13], [14], [15], [16], [17], [18], [19].

We used an eight-color flow cytometry panel combined with TruCount™ beads (for cell enumeration) to create “Flow Count,” a single platform assay for the determination of both the proportion and the absolute count of the major lymphocyte populations (CD4+ and CD8+ T cells, B cells, and NK cells) and that of monocytes and granulocytes. Flow Count was validated on whole blood samples of forty HIV-infected and ten HIV-uninfected subjects by comparison to standard clinical laboratory methods (described in Supporting Information, Text S1 and Figure S1). The relative frequencies and absolute counts of each cell population were determined in paired CSF and peripheral blood samples from both groups. To discover whether differentially effective levels of ART may alter the cellular composition of CSF in HIV infection, we compared the proportions and the absolute counts of CSF cell subsets between subjects on ART, with and without plasma viral suppression, and in subjects off therapy. This report defines both the proportion and absolute number of the CSF B cell, NK cell, and monocyte populations, in addition to the more abundant T cell populations in HIV-infected and uninfected individuals.

## Materials and Methods

### Ethics Statement

This study involving human subjects was conducted according to the principles expressed in the Declaration of Helsinki. Protocols were approved by the UCSF Committee on Human Research and written informed consent was obtained from all study participants.

### Study Design and Participants

This was a cross-sectional study exploring CSF WBC phenotypes in a subset of subjects enrolled in the Sentinel Neurological Cohort (SNC) at the University of California San Francisco (UCSF) [1], [6], [20], [21]. Fifty total subjects were included from four neuroasymptomatic subject groups: (1) HIV-infected subjects taking no ART for at least three months (Offs); (2) HIV-infected subjects on stable combination ART for at least three months with plasma HIV RNA levels >500 copies/mL (Rx Viremic); (3) HIV-infected subjects on stable combination ART for at least 3 months but with plasma HIV RNA levels <500 copies/mL (Rx VL<500) and (4) healthy HIV-uninfected subjects (HIV−).

### Study Procedures, Background Measurements and Virology

CSF was obtained by lumbar puncture (LP) and blood by phlebotomy for study purposes as previously described [1], [20]. HIV RNA concentrations were measured in cell-free CSF and plasma by the Roche Amplicor HIV Monitor assay (versions 1.0 and 1.5, Roche Diagnostic Systems, Inc., Branchburg, NJ) as described [1], [20], [21]. Blood T lymphocyte counts and CSF cell counts were performed at the San Francisco General Hospital Clinical Laboratories using standard methods.

### Flow Count Assay

#### Sample processing

Paired whole blood and CSF samples from each subject were stained in BD TruCOUNT™ tubes (BD Biosciences, San Jose, CA) for cell quantification using a “no wash” procedure to preserve cell numbers. For whole blood, antibodies were combined with 50 µL of whole blood and incubated for 20 minutes at room temperature in the dark. Erythrocytes were then lysed with 450 µL of 1X FACS Lysing Solution (BD Biosciences) and incubated for 10 minutes at room temperature. Control samples (described below) were processed in parallel without TruCOUNT™ beads. Nine milliliters of CSF was collected in a 15 mL tube and processed at 4°C. The CSF was gently vortexed to liberate cells attached to the polypropylene walls of the tube, then centrifuged at 250 *g* for 10 minutes to concentrate the cells. The supernatant was aspirated to 100 µL, and cells were re-suspended in this residual volume before transfer to a TruCOUNT™ tube for staining. CSF cells were stained in the dark for 20 minutes then re-suspended in 350 µL of 0.5% formaldehyde.

Antibodies. CSF and whole blood samples were stained for Flow Count, with the following antibodies: anti-CD45 conjugated with FITC; anti-CD16 and anti-CD56, both conjugated with PE; anti-CD14 conjugated with APC; anti-CD8 conjugated with APC-Cy7; anti-CD4 conjugated with PE-Cy7; anti-CD3 conjugated with Pacific Blue (all from BD Biosciences) and anti-CD19 conjugated with PE-Texas Red (Immunotech, Marseille, France).

Controls. Unstained blood, as well as unstained and single stained BD™ CompBeads (BD Biosciences) controls were prepared for optimizing fluorescence compensation settings for flow cytometric analyses. Fluorescent minus one (FMO) controls were prepared on blood samples by omitting one antibody and used to evaluate fluorescence spill-over and assist with setting gates.

#### Flow cytometry and data analysis

**Figure 1:**
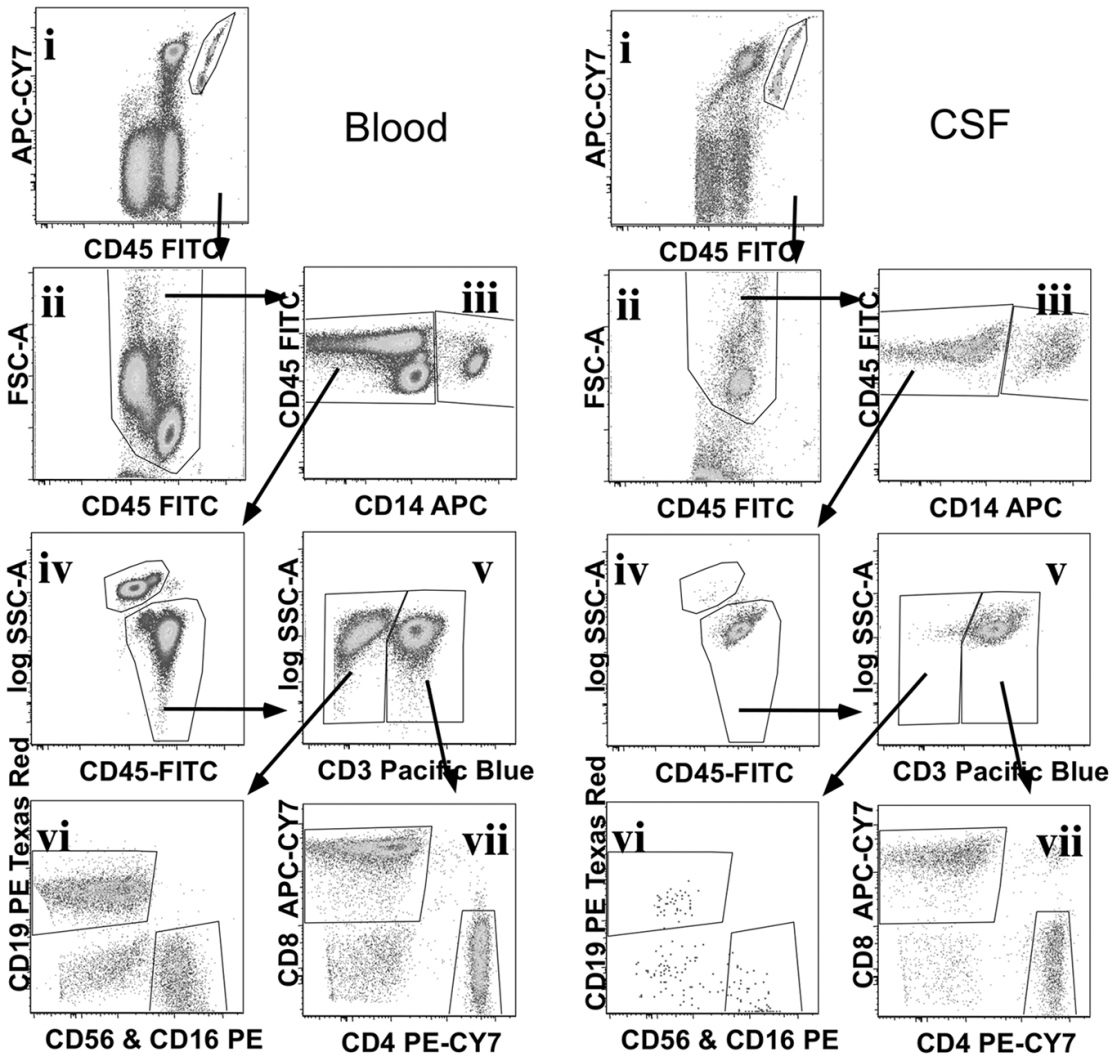
Flow cytometry gating strategy to define blood and CSF white blood cell (WBC) populations. Data was compensated and gated using FlowJo to define TruCOUNT™ beads, (i) from both blood (left panel) and CSF (right panel) data. Remaining events were displayed in a CD45 vs FSC plot (ii) to gate out debris and define a total WBC population. CD14 was used to gate monocytes (iii) from the total WBC gate, and CD14− cells were displayed on a CD45 vs SSC plot (iv) to define lymphocyte and granulocyte gates. Lymphocytes were subdivided into CD3+ T cells and CD3− lymphocytes (v), CD3− lymphocytes were displayed on a CD19 vs CD56&16 (vi) plot to define B cells and NK cells respectively. CD3+ T cells were displayed on a CD4 vs CD8 plot (vii) to define CD4+ and CD8+ T cells.

Stained samples were acquired on a customized LSRII flow cytometer (BD Biosciences) within 2 hours of staining. For blood samples, 100,000 events were acquired; for CSF samples, the entire cell suspension was acquired. Threshold fluorescence was set on CD45-FITC plotted against Side Scatter (SSC) to discriminate the CD45^–^ (debris and red blood cells) population. BD™ CompBeads were stained with the individual antibodies used to stain the cells and run on the same settings. Analysis and compensation of data was performed using FlowJo (Tree Star, Inc., Ashland, OR). The gating strategy used to define each population (Figure 1) was developed on blood and applied to CSF. Adjustments necessary for CSF gating are described below. A plot of CD45 versus APC-CY7 was used to gate and exclude beads. Then CD45 was plotted against Forward Scatter (FSC) and a total WBC gate was drawn to exclude debris. From the total WBC, a plot of CD14 versus CD45 was used to define a gate for monocytes (CD45+CD14+) and lymphocytes plus granulocytes (CD45+/lowCD14low). Lymphocytes and granulocytes were then each gated separately in a CD45 vs SSC plot. From the lymphocyte gate, CD3+ and CD3− populations were gated. The CD3+ population was then gated for CD4+ and CD8+ T cells. The CD3− population was gated for CD19+ B cells and CD56+ or CD16+ NK cells. FMO controls were used to define the position of each gate. The “no wash” procedure used in this study resulted in increased background staining in the CSF sample for some antibodies. This was most pronounced for CD3 staining of monocytes. While blood monocytes were clearly CD3−, CSF monocytes had a low to intermediate level of CD3 staining. We therefore defined monocytes without regard to their level of CD3 expression, in order that the same gates could be applied to CSF and blood. CD3 staining intensity was slightly lower in CSF T cells requiring shifting of CD3 gate to define CD3+ T cells. CD8 staining was higher in CSF, requiring shifting of the CD8 gate to define CSF CD8+ T cells. In addition to measuring the percentage of each gated population, TruCOUNT™ beads were used to determine the absolute number of events per ml of blood or CSF. Absolute counts were calculated by dividing the cell population event count by the TruCOUNT™ bead event count, then multiplying by the ratio of known bead input divided by the original blood (50 µL) or CSF (9 mL) volume.

### Statistics

Statistical analysis was performed using Prism 5 (GraphPad Software, San Diego, CA). Non-parametric tests were used to compare group differences, either Kruskal-Wallis test to compare multiple groups with Dunn’s post hoc comparison within the analysis or Mann-Whitney U-tests to compare two groups. Linear regressions were used for graphic display to compare proportions of CD4+ and CD8+ T cells present in the blood and CSF.

## Results

### Subject Characteristics

Clinical characteristics of the 10 HIV-uninfected and 40 HIV-infected (17 Offs, 12 Rx Viremic, and 11 Rx VL<500) study participants are described in Table 1. All four groups were similar with respect to age, gender, and education. Group differences related to ART treatment history were similar to those described for the larger cohort [1] as were CSF WBC counts that were elevated in the Off group only. None of the subjects suffered ongoing neurological disease. Consistent with our previous report [1], the CSF HIV RNA levels were significantly lower in the Rx Viremic subjects compared with the Offs (difference in medians of 1.50 log_10_, P<0.001) while plasma HIV RNA levels were not significantly different between these 2 groups. The peripheral blood CD4 concentrations were not significantly different among the three HIV-infected groups.

**Table 1:** Background Characteristics of Study Subjects.

	Subject Groups				
	HIV Negative	All HIV-infected	Off Rx	Rx Viremic	Rx VL<500
**Subject Number**	10	40	17	12	11
**Male**	10	38	15	12	11
**Age,** mean +/− SD	47.5+/−6.3	45+/−5.0	42.5+/−5.7	46.3+/−4.8	47.5+/−7.7
**Blood T cells,** Median (IQR)					
**CD4+ cells/µl**	909 (609–919)	251 (179–354)	282 (236–442)	211 (139–250)	256 (172–576)
**CD8+ cells/ul**	406 (338–513)	962 (704–1149)	900 (750–1033)	1018 (611–1268)	1069 (789–1132)
**CD4:CD8 ratio**	1.85 (1.48–2.26)	0.29 (0.18–0.46)	0.36 (0.29–0.51)	0.16 (0.14–0.25)	0.39 (0.21–0.54)
**HIV RNA,** log10 copies/ml, Median (IQR):					
**Plasma HIV RNA**	NA	4.05 (1.83–4.88)	4.41 (4.00–4.89)	4.72 (3.71–5.25)	1.28 (1.28–1.76)
**CSF HIV RNA**	NA	3.02 (1.28–3.60)	3.38 (3.07–4.55)	2.88 (1.61–4.28)	1.28 (1.28–1.28)
**CSF WBCs,** Median (IQR):					
**Cells per ul**	1 (0.0–3.0)	3 (1.0–8.0)	5 (2.0–11.0)	2.5 (1.0–9.5)	1 (0.0–3.0)

SD = standard deviation.

IQR = interquartile range.

NA = Not applicable.

### Lymphocytes are the Predominant WBC Population in the CSF of both HIV-infected Subjects and Healthy Controls

Measurement of lymphocytes, granulocytes and monocytes in the CSF showed that in both HIV-infected and uninfected groups the predominant population was lymphocytes. Monocytes were a minor population (Table 2) and granulocytes were negligible (data not shown). The increased WBC count in HIV-infected subjects was comprised of a 2.8-fold increase in the absolute number of CSF lymphocytes (p = 0.019) and a more modest 1.8-fold increase in absolute CSF monocytes to a level that was not significantly different from the HIV-uninfected group. In contrast, the proportions of CSF lymphocytes and monocytes did not differ between the HIV-infected and uninfected groups (Table 2).

**Table 2:** Proportion and Absolute Count of CSF Populations.

	HIVNegative	All HIV-infected	Fold-increase	p-Value*
	**% of WBC** Median (Range)			
Monocytes	8.44(2.66–25.3)	6.46(1.34–31.9)		0.291
Lymphocytes	89.8(71–95.7)	91.05(64.7–98)		0.52
	**Count per ml of CSF** Median (Range)			
Total WBC	968(413–2616)	2815(338–43944)	2.91	**0.013**
Monocytes	76(17–265)	138(14–932)	1.82	0.099
Lymphocytes	898(326–2352)	2563(269–43035)	2.82	**0.019**
	**% of Lymphocytes** Median (Range)			
CD3+ T cells	94.85(85.7–97.4)	95.5(86–99.2)		0.452
CD4+ T cells	67.16(46.5–77)	21.65(2.47–48.8)		**<0.001**
CD8+ T cells	22.17(11.5–42.3)	60.98(40.4–85.1)		**<0.001**
B cells	0.68(0.27–2.17)	1.21(0.16–8.27)		0.063
NK cells	1.48(0.88–3.44)	1.46(0.22–6.31)		0.707
	**Count per ml of CSF** Median (Range)			
CD3+ T cells	851(311–2238)	2417(231–41132)	2.84	**0.021**
CD4+ T cells	598(152–1498)	458(19–11249)	0.77	0.376
CD8+ T cells	197(46–576)	1672(150–24351)	8.49	**<0.001**
B cells	6.45(1.16–34)	20.53(0.82–1165)	3.18	**0.009**
NK cells	14.15(5.2–40)	29(1.8–406)	2.05	**0.039**

* calculated using Mann-Whitney U-test.

### Distinct CSF CD4+ and CD8+ T Cell Relationships Are Detected in HIV-infected and Uninfected Subjects

**Figure 2:**
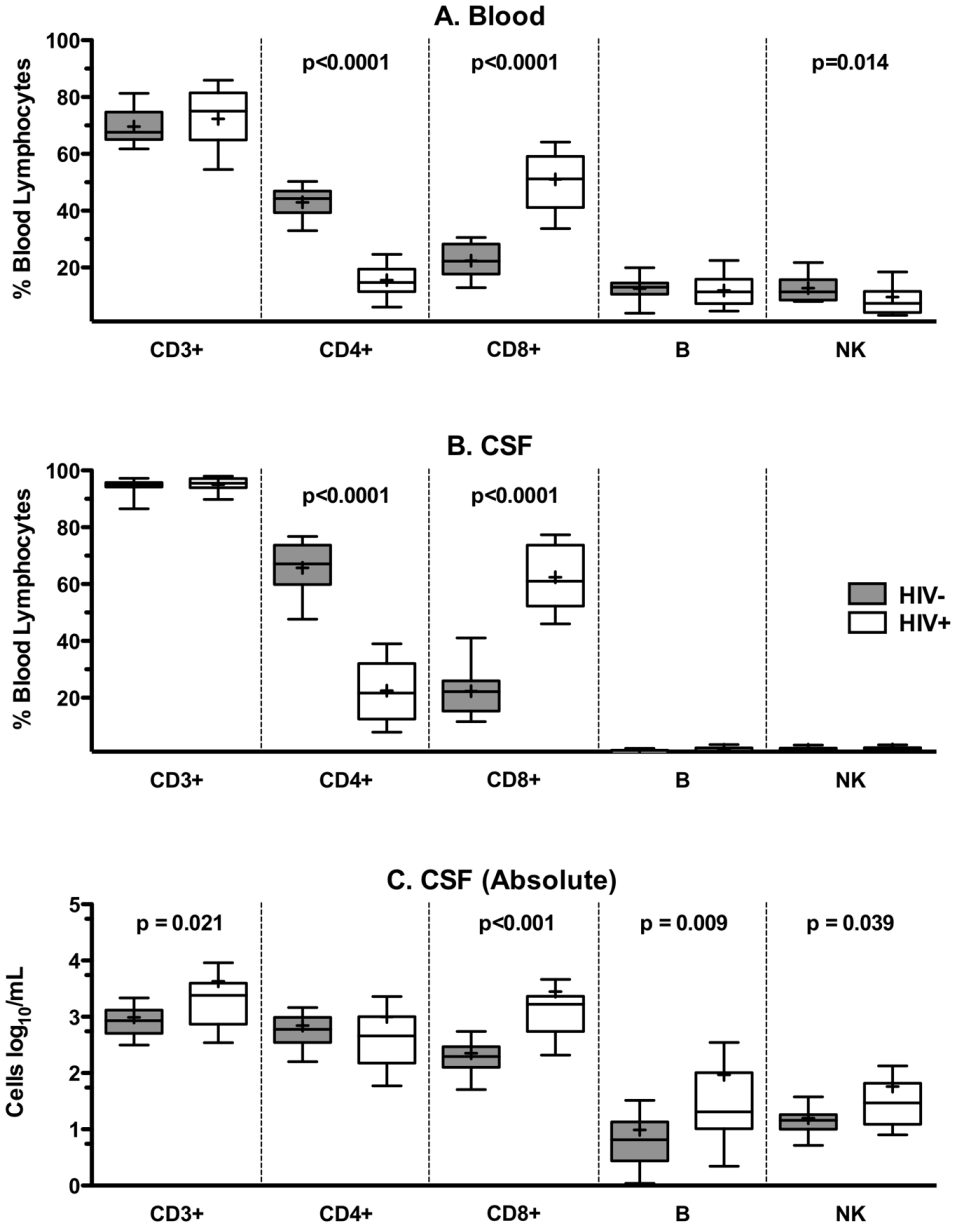
HIV-infected and uninfected subjects have distinct blood and CSF lymphocyte proportions and counts. Proportions of CD3+, CD4+ and CD8+ T cells; B cells; and NK cells present in the blood (A) and CSF (B) of HIV-uninfected (HIV-) and HIV-infected (HIV+) subjects are depicted as a percentage of total lymphocyte population. Absolute CSF cell counts of lymphocyte subsets for HIV-uninfected and HIV infected (C) subjects are shown. Median and interquartile range for each cell population are shown in the box plots. Whiskers are set at 10–90%. Mean values are indicated by (+).

The proportions and absolute counts of CSF and blood lymphocyte populations are shown in Table 2 and Figure 2. CD3+ T cells were the predominant CSF lymphocyte population in both HIV-infected and uninfected subjects (Figure 2B–C, Table 2), similar to the peripheral blood (Figure 2A). The CD4+ T cell subset was most frequent in HIV-uninfected subjects while the CD8+ T cell subset was most frequent in HIV-infected subjects (Figure 2B–C and Table 2).

Compared with the HIV-uninfected, HIV-infected subjects had a significantly lower proportion of CSF CD4+ T cells (p<0.001) and a significantly higher proportion of CD8+ T cells (p<0.001) (Figure 2B–C and Table 2). In addition, the HIV-infected group had a 2.84-fold increase in the absolute number of total CSF T cells (p = 0.021) and an 8.49-fold increase in the absolute number of CSF CD8+ T cells (p<0.001) (Figure 2C and Table 2) compared with the HIV-uninfected group. In contrast, the absolute number of CD4+ T cells did not differ significantly between these two groups. The proportions of B and NK cells were very low in the CSF compared with blood and were not significantly different between the HIV-uninfected and HIV-infected groups (Figure 2A–C and Table 2). However, there was a three-fold increase in absolute CSF B cell count (p = 0.009) and a two-fold increase in CSF NK cell count (p = 0.039) in HIV-infected patients compared with uninfected controls (Figure 2C and Table 2).

### Effect of ART on CSF Cellular Populations in HIV Infection

**Figure 3:**
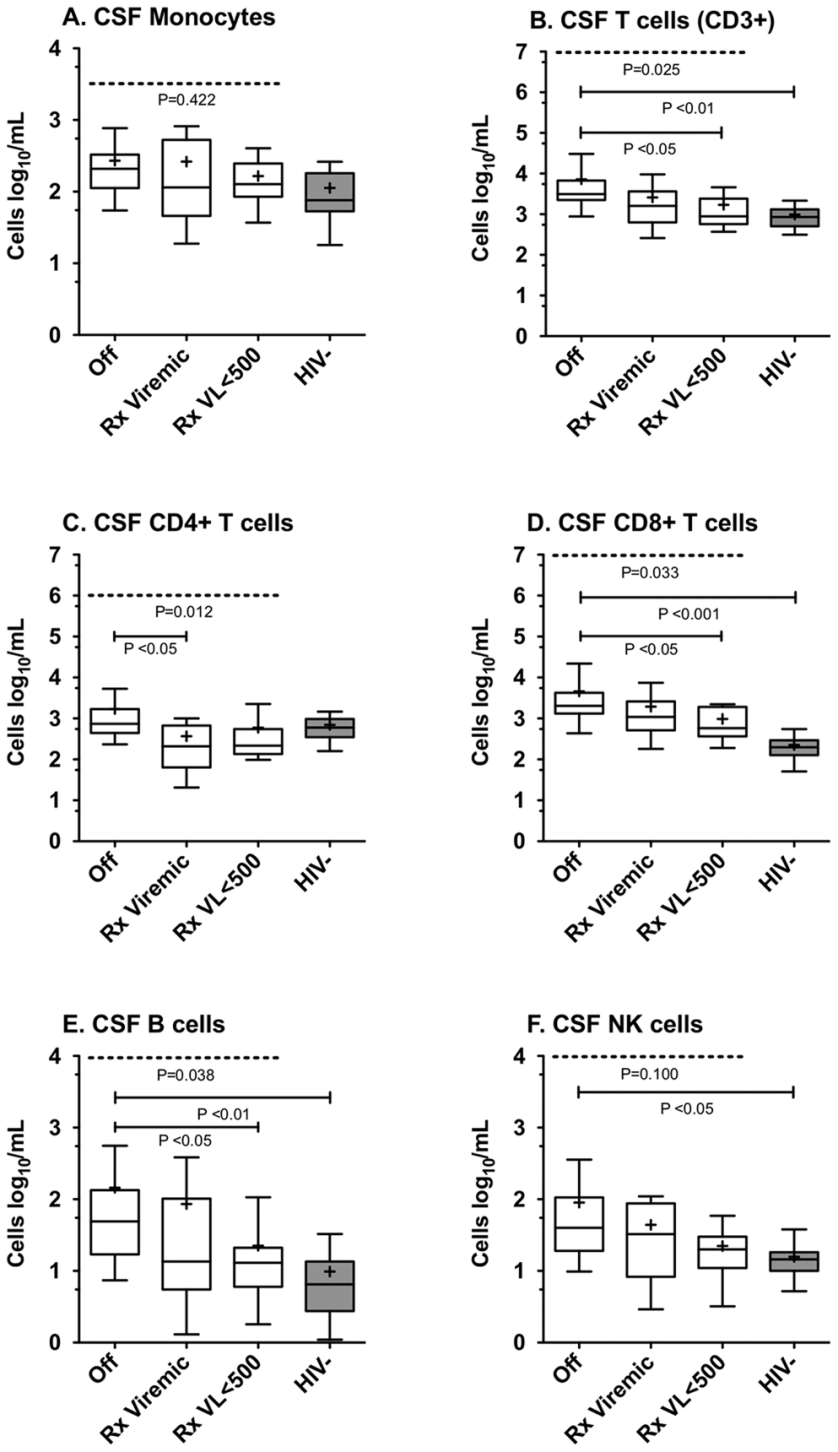
ART influences the absolute lymphocyte subset counts present in the CSF of HIV-infected subjects. Absolute number of CSF monocytes (A), CD3+ T (B), CD4+ T (C), CD8+ T (D), B (E) and NK (F) cell subsets present in CSF of the 4 patient subgroups *(Off, Rx Viremic, Rx VL<500* and *HIV-uninfected)* are shown. Median and interquartile range for each cell population are shown in the box plots. Whiskers are set at 10–90%. Mean values are indicated by (+). ANOVA for significant overall differences between the 3 HIV-infected treatment groups was conducted, with the p value depicted at the top of each panel. Post-hoc analysis using Dunn’s test comparing differences between pairs of groups, if statistically significant, are as indicated in each panel.

The absolute counts of CSF cell populations were compared across HIV-infected subjects grouped according to ART treatment and their response to treatment (Figure 3A–F). There was a significant elevation in CD8+ T cell (10.2-fold) and B cell (7.5-fold) counts in the CSF of HIV-infected subjects off ART compared with HIV-uninfected subjects, while NK cells and monocytes were more modestly increased (2.8- and 2.7-fold), similar to total T cells (3.6-fold).

Within the three HIV-infected groups, treatment was associated with lower CSF T cell and B cell, but not CSF monocyte or NK counts (Kruskal-Wallis; Figure 3). CD8+ T cell and B cell counts were both significantly higher in subjects off ART compared to those on virologically suppressive treatment (p<0.05, Dunn’s post-test; Figure 3D–E). We expected to see the opposite trend for CSF CD4+ T cells, namely increased CD4+ T cell counts in the group on virologically suppressive treatment compared with the Off group; however CSF CD4+ T cell counts were not significantly different between these two groups, but were significantly higher in the Off treatment group compared with incomplete viral suppression (p<0.05, Dunn’s post-test; Figure 3C).

### Relationships between Blood and CSF Cellular Populations in HIV-Infected Subjects

**Figure 4:**
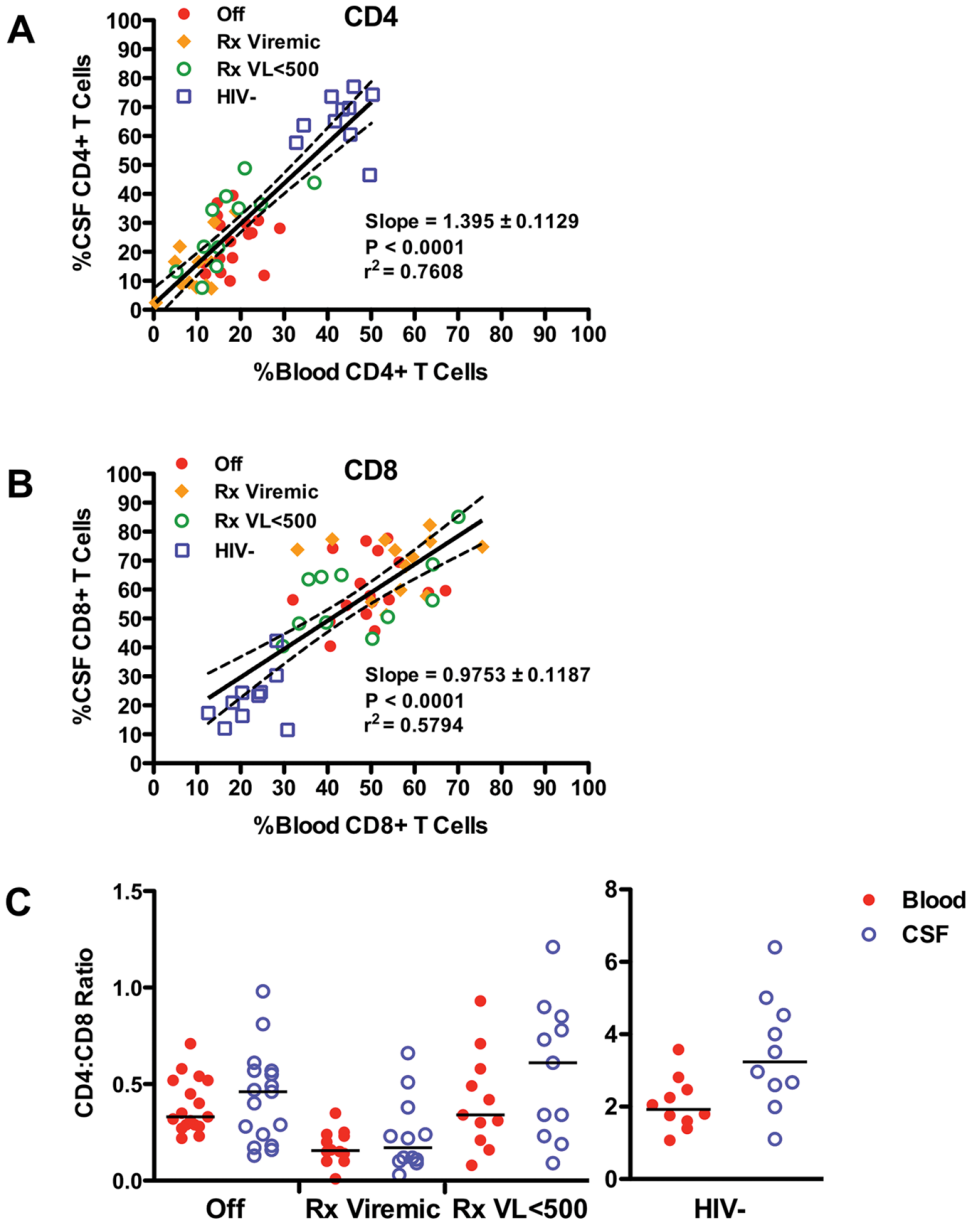
Comparison of T cell subsets in CSF and blood of HIV-infected and uninfected subject. (A) Relation of CD4+ T cells present in the CSF to those present in the blood amongst the 4 subject groups with regression line (solid line; slope  = 1.395, r^2^ 0.7608, p<0.0001) and 95% confidence intervals (dotted lines). (B) Relation of CD8+ T cells present in the CSF to those present in the blood amongst the 4 subject groups with regression line (slope  = 0.9768, r^2^ 0.5794, p<0.0001) and 95% confidence intervals. (C) Comparison of CSF and blood CD4:CD8 ratio between the 4 subject groups.

Since the pattern of CD4+ and CD8+ T cell changes in CSF of HIV- infected subjects were similar to those observed in the blood, linear regression was used to examine the relationship between blood and CSF cell populations. As shown in Figure 4, the proportion of both CD4+ and CD8+ T cells in the CSF correlated significantly with their respective proportions in the blood (CD4: r^2^ = 0.7608, p<0.0001; CD8: r^2^ = 0.5794, p<0.0001). In contrast, CSF NK cell, B cell, and monocyte counts did not show significant correlation with the corresponding populations present in the blood (data not shown).

To better understand the pattern of CSF CD4+ and CD8+ T cell changes across HIV-infected groups, we examined the CSF and peripheral blood ratios of CD4+ to CD8+ T cell absolute counts (CD4:CD8) (Figure 4C). In HIV-uninfected subjects the CD4:CD8 ratio was significantly higher in the CSF than the blood (median 3.2 versus 1.9; p = 0.0232) while in the viremic HIV-infected groups, CD4:CD8 ratios of blood and CSF were similar (Rx Viremic: median 0.17 in CSF and 0.15 in blood; Offs: median 0.46 in CSF and 0.33 in blood). In the Rx VL<500 group, the CSF CD4:CD8 ratio was increased in comparison to blood, similar to HIV-uninfected controls (median 0.61 versus 0.34), but this difference was not significant.

### Relationship Between CSF HIV RNA Level and CSF Cell Populations in HIV-infected Subjects Off ART

Since CSF WBC count has previously been shown to correlate with CSF HIV RNA level [4], [6], we examined the relationship between HIV RNA level and the absolute counts of each CSF cell population in the Off ART group, since this group had measurable CSF RNA copy numbers. Within this group, CSF HIV RNA level correlated strongly with total WBC (p<0.0055), total lymphocyte count (p<0.0051), CD8+ T cell (p = 0.0062), CD4+ T cell (p = 0.0103), and B cell counts (p<0.0042), but not with NK cell counts (p<0.0892) or the absolute count of monocytes (p<0.2808).

## Discussion

Flow cytometry has become widely used for analysis of the cellular content of CSF, and the utility of this technique for samples with low numbers of cells has increased as advances in technology have allowed more parameters per individual cell to be interrogated [12]. We developed and validated a quantitative flow cytometry assay (Flow Count) in comparison with clinical laboratory tests (CBC-diff and MultiTest, described in Supporting Information, Text S1 and Figure S1) and used it to quantify both the absolute count and the proportion of the major WBC populations in the CSF of HIV-uninfected and HIV-infected subjects. Absolute counts revealed differences that were not apparent on examination of cell proportions alone, allowing a more thorough evaluation than previous studies [7], [14]. In contrast to most previous studies of CSF T cells [10], [15], [16], [18], [22], [23], our healthy control subjects consisted of truly neuroasymptomatic HIV-seronegative donors, rather than subjects with non-inflammatory neurological disease (NIND).

We found that in healthy controls the cellular composition of the CSF differs from whole blood; as previously reported in healthy controls and subjects with NIND, granulocytes are rare and CD4+T cells are the predominant population in CSF [7], [15], [23]. The relative enrichment of CD4+ T cells in the CSF compared to CD8+ T cells, NK cells and B cells most likely reflects the role of CD4+ T cells in immune monitoring and suggests that in the healthy state, CD4+ T cells can preferentially cross the blood-CSF barrier [24]. In HIV-infected subjects there was a generalized increase of all lymphocyte populations, except CD4+ T cells, and a massive increase in both the proportion and absolute number of CD8+ T cells suggesting that lymphocyte trafficking to the CSF is dramatically altered with HIV infection.

CSF pleocytosis occurs early in HIV infection and in the absence of opportunistic brain infection [3], [4], [25], [26], [27]. In untreated chronic infection, the CSF WBC count correlates with CSF and plasma HIV RNA levels and with the level of blood and CSF CD8+ T cell activation [11], [17], [28], [29], [30]. Transmission of HIV into the CSF compartment and its amplification therein, via infected CD4+ T cells and monocytes, is facilitated and maintained by this pleocytosis. We have previously proposed a “push-pull” model to explain transmigration of T cells to the CSF compartment in HIV infection [9]. In simple terms, activation of peripheral cells results in increased ability to cross the blood-CSF barrier (push), while intrathecal HIV-infection and consequent release of inflammatory chemokines attracts and retains more cells (pull). Both CD4+ and CD8+ T cells likely increase their rate of transmigration in response to both peripheral and intrathecal inflammation and we have previously demonstrated increased peripheral and CSF levels of CD8+ T cell activation in this cohort [11]. In addition, cellular proliferation in response to inflammatory signals, retention within the CSF in response to recognition of cognate antigen, and killing of CD4+ T cells as a consequence of HIV infection may also occur within the CSF.

We found that the ratio of CD4+ to CD8+ T cell absolute counts (CD4:CD8) in HIV-uninfected subjects was higher in the CSF than the blood consistent with previous reports for normal controls and subjects with non-inflammatory neurological disease (NIND) [7], [22]. In the absence of peripheral inflammation, CD4+ T cells may be more likely to traffic to the CSF than CD8+ T cells, or alternatively, may be retained or survive for longer in the CSF compartment than CD8+ T cells**.** Reversal of the CD4:CD8 ratio in the CSF of HIV-infected subjects is consistent with a previous study [14], which suggested that in untreated HIV-infection, T cell phenotypes in CSF are largely determined by the corresponding proportions in the peripheral blood. We found an overall correlation between CSF and blood proportions of both CD4+ and CD8+ T cells and in comparison with the HIV-uninfected group, the CSF CD4:CD8 ratio of subjects in the ART suppressed group was decreased to the same extent as the blood CD4:CD8 ratio. However the CSF CD4:CD8 ratio in viremic subjects was more substantially decreased than the blood CD4:CD8 ratio, in comparison to HIV-uninfected subjects, suggesting a disproportionate CD8+ T cell increase in the CSF of viremic patients compared with blood (CSF CD4+ T cell counts were not usually decreased). In addition, CSF CD8+ T cell counts in the Off therapy group were strongly correlated with CSF HIV RNA level. Together these data suggest that while changes in peripheral blood T cells are a strong determinant of CSF cell content, HIV-induced inflammatory signaling may contribute to CD8+ T cell transmigration, retention or proliferation in patients that are viremic.

B cells were extremely rare in the CSF, as previously reported in normal and NIND subjects [7], [23], [33]. Their frequency was significantly increased with HIV infection and correlated with CSF viral load in agreement with a previous report comparing B cells in HIV-infected and NIND subjects [33]. However, the observed increase in CSF B cells with HIV-infection was considerably less than previously reported for other inflammatory diseases such as multiple sclerosis and neuromyelitis optica, [8], [18], [19], [34], [35] or CNS infection, such as Lyme disease or neurosyphilis [36], [37]. The presence of IgG in the CSF of a majority of HIV-infected individuals [38] suggests that local B cell immune responses occur in response to HIV infection of the CNS. Thus, the changes we observed in response to HIV-infection and treatment may reflect a B cell response to intrathecal HIV replication.

While the absolute number of CSF WBC, lymphocytes, and T cells in our study was very similar to a previous report on the frequency of these cells in the CSF [23], the frequency of monocytes in normal CSF was 3-fold lower in our study (although our values were in the range detected in the previous study). Potential explanations include differences in patient populations, sample processing and gating strategies. General inflammation associated with stress may have increased circulating and CSF monocytes in the previous study where normal CSF samples were obtained from individuals undergoing surgery, while in our study the HIV-1 negative samples were obtained from healthy volunteers. In the previous study, CSF was collected into a stabilization buffer and processed within six hours, while we stained concentrated cells in CSF within one hour of collection, an approach that has worked well for ]ymphocyte studies but may not be optimal for the survival of CSF monocytes (reviewed in [39]). Monocytes were identified by CD45 staining and side scatter in the previous study, which could potentially over-estimate monocyte numbers; our more stringent gating strategy may have excluded some cells (Supporting Information, Text S1). Further studies are necessary to determine an optimal procedure for processing and staining of CSF monocytes and to establish a normal range for these cells in the CSF.

NK cell frequencies in the CSF have not been reported in HIV-infected subjects. Previous studies of normal CSF reported a very low frequency [7]. Our results confirmed that while NK cell counts are very low in the CSF, cell counts were quite variable. This variability may reflect changes in peripheral NK cell dynamics. We detected a significant loss of blood NK cells in the subjects off of ART compared with controls, while CSF NK cells were modestly but significantly increased in this group. These data may reflect a redistribution of circulating NK cells to tissues, including the CNS, in response to inflammatory responses to HIV such as chemokine signaling [40] although this variability could also result from differential background staining in CSF compared with blood (Supporting Information, Text S1).

Patients in this study were all neurologically asymptomatic but most had abnormally high levels of CSF CD8+ T cells, even when virologically suppressed, and may be at risk for development of neurological impairment. While ART has drastically reduced the incidence of HIV associated dementia (HAD) [41], [42], milder neurological impairment is reported to remain common [43], [44]. The pathogenesis of this milder impairment is not well understood; it is not yet clear whether this reflects brain injury sustained before treatment, or ongoing indolent injury. Impaired performance has been correlated with lower blood CD4 nadir [45], which might be compatible with both of these explanations. Thus, prior reduction of blood CD4+ T cells, as a surrogate for systemic disease progression, might also indicate a longer and more severe course of CNS infection and attendant injury related to immune-mediated neuropathology [46]. Since CD8+ T cell activation associates with both pretreatment CSF HIV-1 RNA levels and the CD4 nadir, the later may indicate prior activation, CNS infection and susceptibility to virus-driven CNS injury. However, the persistence of high numbers of CD8+ T cells and CD8+ T cell activation even after treatment [11] might also indicate and perhaps contribute to continued CNS injury, and the lower CD4 nadir may also indicate a predisposition to continued immune activation and subsequent ongoing CNS injury. Whatever the mechanism, the continued presence of large numbers of activated CD8+ T cells in CSF indicates a perturbation in CNS immunological homeostasis that is partially though incompletely ameliorated by treatment. These considerations raise the question of whether early treatment, which has been shown to reduce CD8+ T cell activation in the periphery [Jain, unpublished observation] might more effectively prevent the development of this state and reduce the effects of chronic CNS immunoactivation that may underlie neurological impairment.

In conclusion, our study provides a comprehensive analysis of all the major WBC populations in the CSF of neuroasymptomatic HIV-negative healthy controls and HIV-infected subjects using Flow Count, a polychromatic flow cytometry method. This report provides novel insight into CSF cell populations counts, and into the relationship between CSF and blood cellular composition in different settings of HIV infection and treatment. Our results indicate that even in the setting of ART, an increase in CD8+ T cells, as well as a generalized increase in most other cell types present in normal CSF, characterizes HIV infection. Whether the less frequent NK, B cell and monocyte populations are subject to the same factors that induce transmigration of T cell is not clear, but they may play an important role in the initiation and maintenance of HIV infection of the CSF and brain (monocytes), in control of that infection (NK and B cells), and in the production of potentially neurotoxic inflammatory agents. Given that a significant percentage of HIV-infected patients may have worsened cognition [43] even while on ART, further studies to assess the phenotypes and function of cell populations in untreated and ART-treated HIV infection are warranted to provide further insight into the pathogenesis of neurologic deficits identified in the current era.

## Supporting Information

Figure S1 **Comparison of cell quantification by standard clinical laboratories and flow cytometry-based assay.** Correlation of the standard clinical laboratory and the Flow Count method (Left column) and standard difference between paired results of each assay, plotted against the mean of the paired results using a Bland-Altman plot (right column). Mean bias between the 2 assays is shown as a solid line and the 95% limits of agreement is shown as a dotted line.
(TIFF)

Text S1 **Validation of Flow Count Assay.**
(DOCX)
