# Supplementary figures and images for: Cellular Composition of Cerebrospinal Fluid in HIV-1 Infected and Uninfected Subjects
Source: PLoS One. 2013 Jun 18;8(6):e66188. doi: 10.1371/journal.pone.0066188 (PMC3688831; doi:10.1371/journal.pone.0066188)

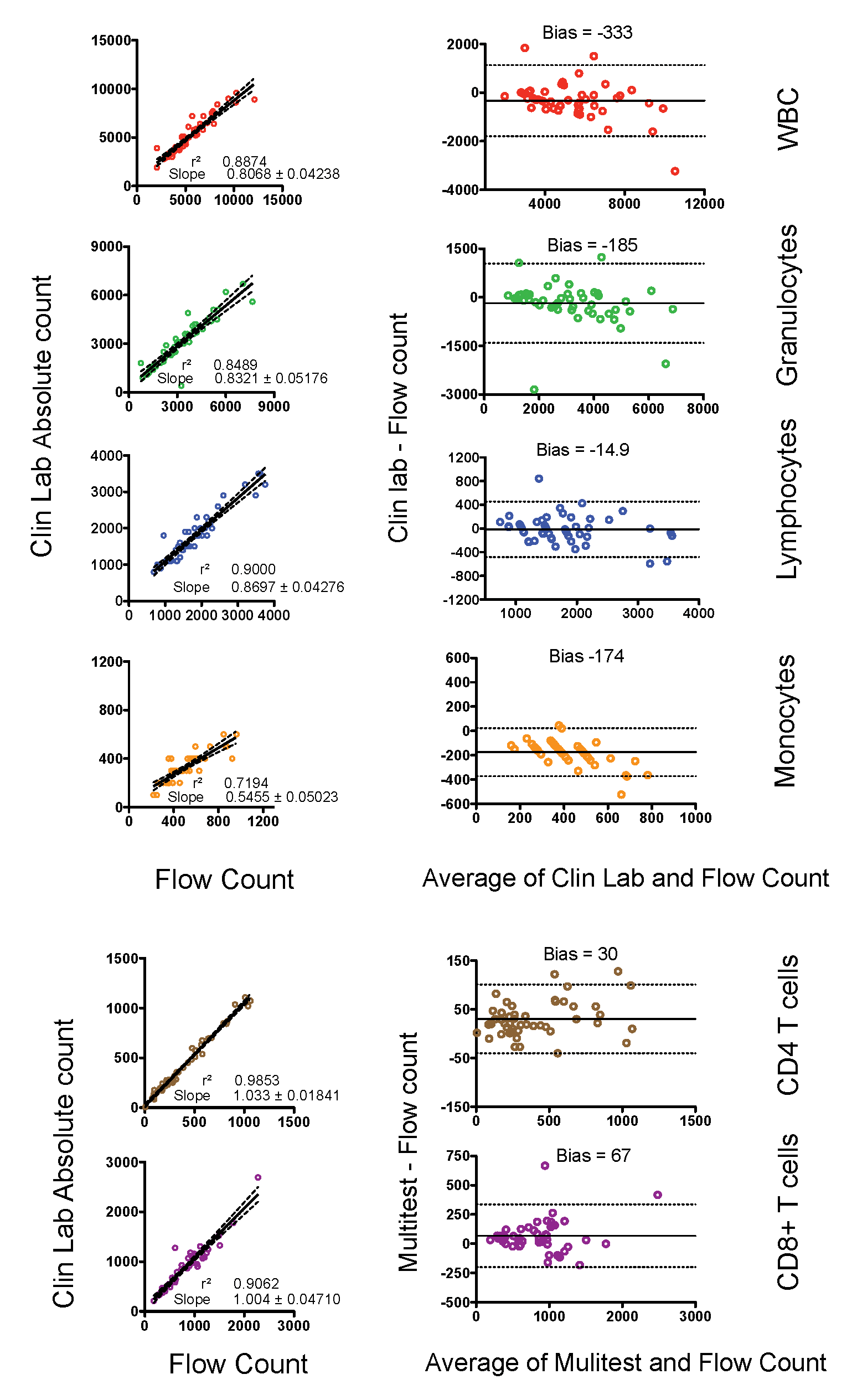

Supplement: Figure S1 — Comparison of cell quantification by standard clinical laboratories and flow cytometry-based assay. Correlation of the standard clinical laboratory and the Flow Count method (Left column) and standard difference between paired results of each assay, plotted against the mean of the paired results using a Bland-Altman plot (right column). Mean bias between the 2 assays is shown as a solid line and the 95% limits of agreement is shown as a dotted line. (TIFF) [file pone.0066188.s001.tiff]
