# Supplementary material for: Cellular Composition of Cerebrospinal Fluid in HIV-1 Infected and Uninfected Subjects
Source: PLoS One. 2013 Jun 18;8(6):e66188. doi: 10.1371/journal.pone.0066188 (PMC3688831; doi:10.1371/journal.pone.0066188)
Supplement: Text S1 — Validation of Flow Count Assay. (DOCX) [file pone.0066188.s002.docx]

#### Validation of “Flow Count” for White Blood Cell Count and Differential.

The 8-color Flow Count assay applied to whole blood was compared to results of standard clinical laboratory complete white blood cell and differential (CBC-diff) counts. We found very strong correlation between the Flow Count assay and the CBC-diff counts for total white blood cells (WBC), lymphocytes and granulocytes (Figure S1, all p>0.0001) from whole blood. Although there was a strong correlation between peripheral blood monocyte counts in the two tests (p<0.0001), monocyte numbers were significantly higher in the Flow Count assay than the Clinical Laboratory CBC-diff. We also tested for numeric agreement between clinical laboratory and Flow Count results. The differences between the paired results of each assay were plotted against the mean of the paired results in Bland-Altman plots (Bland and Altman, 1986). The mean bias between the CBC-diff and Flow Count data and the 95% limits of agreement are as shown (Figure S1, right panel). A mean bias greater than zero indicates that the clinical laboratory results were higher than the Flow Count results, and the within-subject variability can be assessed by how close the 95% limits of agreement are to the bias. The mean bias between CBC and flow count on whole blood for total WBC was -333 with limits of agreement from -1797 to 1130. In the context of a clinical test with a normal range from 3900 to 11,000, this bias and 95% limits shows very good agreement between the two tests. Good agreement was also observed for granulocytes (mean bias -185 with 95% limits of agreement from -1410 to 1039, normal range 1700 to 8000) and lymphocytes, (mean bias -15, 95% limits of agreement -483 to 454, normal range 1000 to 5100). Monocyte counts showed more variability between the two assays. The mean bias was -174 indicating that the Flow Count results were higher than the CBC results and the limits of agreement were -371 to 22 in the context of a test with a normal range from 0 to 800.

CD4+ and CD8+ T cells counts obtained with the Flow Count assay were compared with the BD Multitest™ assay run by the clinical laboratory. As expected, there was a very strong correlation between Multitest™ and Flow Count results (p>0.0001), since both are flow cytometry-based methods (Figure S1). We also observed strong agreement between the two assays both for CD4+ and CD8+ T cell absolute counts. The mean bias of CD4+ T cells was 30, with 95% limits of agreement from -40 to 101, normal range 420 to 1250. The mean bias of CD8+ T cells was 67 with 95% limits of agreement from -200 to 336, normal range 220-960.

We found strong correlation and agreement between Flow Count and clinical tests, for both the percentages and absolute numbers of whole blood CD4+ and CD8+ T cells, lymphocytes, granulocytes and total WBC. However, there was less agreement between the results of these tests with respect to whole blood monocytes. This is likely due to differences in the methodologies of the 2 assays. While the clinical lab assay relies on peroxidase activity to identify monocytes, the Flow Count assay identifies these cells based on surface expression of CD14. It is possible that the peroxidase method underestimates monocytes, or alternatively, staining with CD14 antibody could overestimate their numbers. For all whole blood populations examined, there was some inter-individual variation in the results obtained by the two assays, indicating that while the Flow Count assay can be used with confidence on a population basis, further validation would be necessary for determination of cell counts on individual patients.

Limitations of Flow Count Assay when Applied To CSF

The Flow Count assay did have several limitations when used with CSF. Since cells had to be pelleted, resuspended and then transferred to TruCOUNT™ tubes for staining, inevitably some loss of cells occurred. In addition, the “no wash” procedure, employed to reduce cell loss, did induce some background staining in CSF cells. While most populations were clearly distinguishable even with increased background, CD14+ monocytes bound a considerable amount of CD3 antibody, which necessitated defining monocytes without regard to CD3 expression. In addition, CSF monocytes had higher CD14 background staining in CSF than blood, requiring a tighter gate, which may have excluded CD14lo non-classical monocytes. This population is infrequent in the CSF (median 1% of total CSF monocytes, unpublished observation) and its exclusion is unlikely to have decreased the detected frequency of CSF monocytes. However, this gating difference may have impacted CSF NK cell numbers, as CSF CD14 lo monocytes, which also express CD16, could potentially have been included in the CD3-CD16+ NK cell gate (Figure 1). Due to the very low frequency of NK cells in normal CSF, even a small number of contaminating monocytes could have increased the measured frequency of this population.

Despite these limitations, the Flow Count assay allowed us to perform a comprehensive analysis of WBC populations in the CSF of HIV-infected and HIV-negative subjects in comparison to matched whole blood samples. This method overcame the limitations of low cell numbers and poor definition of lymphocytes as previously reported by Svenningsson and colleagues, who used 4-color flow cytometery to examine the cellular content of CSF from neurologically normal subjects (Svenningsson et al., 1995). This group had to split CSF samples in order to examine more than 4 parameters, resulting in insufficient cell numbers for some analyses. Since 3,500 to 20,000 cells can be obtained from 9 ml of CSF from a typical donor, the 8-color Flow Count assay allowed us to apply a single panel to all cells. Additionally, Svenningsson and colleagues noted that using only forward and side scatter to define a lymphocyte gate was inaccurate in CSF, as debris and red blood cells could overlap with this gate (Svenningsson et al., 1995). In the present study, we used CD45 to define WBCs (including lymphocytes), effectively excluding debris and occasional red blood cells. The 8-color Flow Count assay used in this study was validated on fresh whole blood samples in comparison with clinical laboratory CBC-diff and MultiTest results, demonstrating that absolute counts of all the major white blood cell populations can be obtained by flow cytometry in conjunction with BD TruCOUNT™ beads.

Bland JM, Altman DG (1986) Statistical methods for assessing agreement between two methods of clinical measurement. Lancet 1: 307-310.

Svenningsson A, Andersen O, Edsbagge M, Stemme S (1995) Lymphocyte phenotype and subset distribution in normal cerebrospinal fluid. J Neuroimmunol 63: 39-46.
